# Patterns of Recombination in Coronaviruses

**DOI:** 10.3390/ijms26125595

**Published:** 2025-06-11

**Authors:** Ricardo Soares, Cristina P. Vieira, Jorge Vieira

**Affiliations:** 1 Instituto de Investigação e Inovação em Saúde (i3S), Universidade do Porto, Rua Alfredo Allen 208, 4200-135 Porto, Portugal; ricardo.soares@i3s.up.pt (R.S.); cgvieira@i3s.up.pt (C.P.V.); 2 Instituto de Biologia Molecular e Celular (IBMC), Rua Alfredo Allen 208, 4200-135 Porto, Portugal; 3 School of Medicine and Biomedical Sciences (ICBAS), Porto University, Rua de Jorge Viterbo Ferreira 228, 4050-313 Porto, Portugal; 4 Faculdade de Ciências da Universidade do Porto (FCUP), Rua do Campo Alegre s/n, 4169-007 Porto, Portugal

**Keywords:** *Coronaviridae*, recombination, RDP5, selection

## Abstract

By bringing together different variant combinations, recombination can contribute to adaptation in *Coronaviridae* species, some of which infect humans, and have given rise to epidemics and a pandemic. Therefore, in this work, the impact of the use of different recombination inference methods and sample sizes is addressed using data from 21 *Coronaviridae* species, and recombination inferences are further supported using a phylogenetic approach. Recombination patterns are shown not to vary greatly between species. A positive correlation is found between gene position and recombination rates, suggesting intrinsic variation in recombination rates along the genome. Within and between species recombination patterns are shown to differ, the module type being the most prevalent between species except for the *Membrane* and *Nucleocapsid* genes, whose products are known to interact and thus must co-evolve, explaining why the two genes are often recombined as one unit. It is also shown that within species, the module type is prevalent for the *Spike* gene only. Moreover, a positive correlation between recombination and selection is here reported. Therefore, intratypic recombination patterns are also shaped by selection. Recombination may thus be an important source of variability upon which selection can act.

## 1. Introduction

In viruses, variability is essential for the adaptation to different hosts and efficient spreading. Such variability is generated through mutation, recombination, and in segmented RNA viruses (viruses that maintain their genomes as several distinct RNA molecules), through reassortment (the combination of genome segments from different parental origins) [1]. Members of the *Coronaviridae* family are non-segmented membrane-enveloped positive sense ssRNA viruses that infect animals (commonly referred to as Coronaviruses). This family is divided into three subfamilies, namely the *Letovirinae* (one genus only), *Pitovirinae* (one genus only), and the *Orthocoronavirinae* (including the *Alpha-*, *Beta-*, *Delta-*, and *Gammacoronavirus* genera that form distinct monophyletic groups) [2]. Members of the *Betacoronavirus* genus have become human epidemics (SARS-CoV and MERS-CoV) or even pandemics (SARS-CoV-2) [3]. Moreover, four other species of *Coronaviridae* (all belonging to the *Alpha-* and *Betacoronavirus* genera, namely hCoV-229E, hCoV-OC43, hCoV-NL63, and hCoV-HKU1) have been documented to be able to infect humans [3]. Therefore, it is of interest to address the relative contribution of mutation (single nucleotide changes) and recombination (combinatorial changes) to genetic variability in the *Coronaviridae* family.

For within species (intratypic) recombination to happen, two different viruses of the same species need to be present in the same cell, at the same replication point. Evidence for intratypic recombination has been found for the *Alphacoronavirus* hCoV-NL63, and the *Betacoronavirus* species hCoV-OC43, hCoV-HKU1, MERS-CoV, and SARS-CoV-2 [4,5,6,7,8]. Between species (intertypic) recombination is also likely possible, since *Coronaviridae* share a comparable genome organization [9]. The basic organization is from 5′ to 3′ *ORF1ab* (that encodes two nonstructural polyproteins, expressed by the ribosomal frameshifting mode), followed by the structural protein genes *Spike* (*S*), *Envelope* (*E*), *Membrane* (*M*), and *Nucleocapsid* (*N*) [10]. The *Accessory* genes are the only ones that have a more variable positioning within the genome, albeit always following *ORF1ab*. Still, in order for two species to co-exist in the same cell, they must share the same host and tissue tropism [11]. In coronaviruses, given their wide host species range, including bony fishes, amphibians, birds, and mammals, it could be reasoned that intertypic recombination would be rare. Nevertheless, their presence in humans and economically important species, such as livestock and poultry [12,13], may facilitate cross-species transfer. The host specificity is dependent on the receptor used to enter cells, and thus on the binding of the receptor binding domain of the *S* protein [14,15], the main target of the immune system [16]. Nevertheless, there are also reports of receptor independent host cell entry [17,18], which could also facilitate cross-species recombination. Nikolaidis, et al. [19] found evidence for recombination between the SARS-CoV-2 *Betacoronavirus* and other species of the same genus. Moreover, hCoV-HKU1 (*Betacoronavirus*) has also been found to be able to recombine with other *Betacoronavirus* species [20]. Cases of intertypic recombination between species from different *Orthocoronavirinae* genera are likely very rare, and between species from *Alpha-* or *Betacoronavirus* and *Gamma* or *Deltacoronavirus* even rarer, since while all genera include species capable of infecting mammals, *Alpha-* and *Betacoronavirus* do so exclusively, while *Gamma-* and *Deltacoronavirus* primarily infect birds [2]. A single reliable case of intergenus recombination between an *Alphacoronavirus* and a *Deltacoronavirus* was identified so far [21], as well as a single case between a *Gammacoronavirus* possibly recombining with an *Alpha* or *Betacoronavirus* giving rise to the mosaic nature of the SARS-coronavirus genome [22].

The exact details on how recombination happens in viruses are not known, but the main hypothesis is the “copy-choice” replication [23]. It consists of a switch of the template by the RNA-dependent RNA polymerase, by three proposed scenarios, namely, a similarity-essential, a similarity-nonessential, and a similarity-assisted recombination scenario [24]. The first one requires that both recombinant sequences show some degree of homology in order for the RNA-dependent RNA polymerase (RdRp) to switch from one template to another. The second one does not require sequence homology but in turn makes use of other signals, such as the RNA secondary structure, or specific signal sequences, such as transcription regulatory sequences (TRS) [11]. Finally, the third is a mixture of the first two.

Depending on the relative frequency of the different types of events (similarity-essential, similarity-nonessential, and similarity-assisted), recombination patterns may arise in the genome of coronaviruses. However, such patterns may be attenuated by selection. Indeed, recombination has often been regarded as having the potential to introduce variability in coronaviruses’ proteins, with a possible impact on transmissibility and host spillover [25,26,27,28,29]. The finding of a strong recombination signal around the *S* gene, [30,31,32,33], as well as the location of the breakpoints, suggestive of the transfer of this gene as a unit [19,34], supports this view. For instance, despite the low frequency of recombination estimated for SARS-CoV-2 (16 recombinant genomes out of a pool of 279,000 sequences obtained from the end of 2020 to early 2021; [8]), in 10 out of these 16 cases, either the recombinant inherited the *S* of the most prevalent variant (B.1.1.7) or the break point was observed to be near the 5′ end of the *S* gene. However, it should be noted that recombination hotspots have been described near other genes as well, such as the *Accessory* genes [32].

Despite all the available evidence, the role of recombination in the generation of coronaviruses variability is still debatable. For instance, Forni, Cagliani and Sironi [33] found no association between recombination levels (inferred using 3SEQ only) and selection, in contrast to the expected pattern under the hypothesis that recombination is an important source of positively selected amino acid variants. Nevertheless, it could be argued that the ability to infer selection may be dependent on the sample composition and size available for each species, as well as the method used. Therefore, it might be useful to also look at the relationship between selection and recombination along the genome of a given species, for as many species as possible to see if any patterns emerge. Under the assumption that all coronaviruses are under the same selective constraints, a good estimate for the proportion of amino acid positions that are under positive selection at all coronaviruses proteins can be obtained from the work of Ferreira et al. [35]. These authors performed a genome-wide analysis, using 15 *Coronaviridae* species to identify a set of 199 well-supported amino acid positions that could be mapped to SARS-CoV-2 proteins that have been the subject of positive selection (58 in ORF1a, 23 in ORF1b, 54 in S, 1 in E, 11 in M, 32 in N, and 20 in the Accessory proteins). These numbers can be normalized by the protein sizes observed in SARS-CoV-2 reference genome (NC_045512.2) to estimate the percentage of amino acid positions that for a given protein are under positive selection (1.32% for ORF1a, 0.86% for ORF1b, 4.24% for S, 1.33% for E, 4.96% for M, 7.64% for N, 3.03% for the Accessory proteins) and use these as a proxy for the average percentage of amino acid positions under positive selection in coronaviruses in general, as in this work.

Besides recombination inferences using multiple methods, phylogenetic approaches can also be employed to assess recombination in coronaviruses, as recombination leads to incongruent evolutionary histories at different regions of the genome as shown in [36,37]. Moreover, under the assumption that recombination is an important source of variability upon which selection can act upon, a correlation between *Ka* values and recombination has been looked for [20,38,39]. Therefore, in this work, we address the role of recombination and selection in 21 species of the *Coronaviridae* family by performing intratypic (within species) and intertypic (involving more than one species) recombination inferences, applying multiple approaches, phylogenetic inferences, and by looking at non-synonymous variability rates.

## 2. Results

### 2.1. Recombination Inferences

Of all the methods used (3SEQ, Geneconv, Bootscan, RDP, Chimaera, Siscan, and MaxChi, implemented on the recombine (tag 1.0.0) Docker image, available at the pegi3s-Bioinformatics Docker Images Project (BDIP) (http://bdip.i3s.up.pt/container/recombine, accessed on 12 February 2024), only 3SEQ, Geneconv, RDP, and Bootscan reliably produced statistically supported breakpoints, and thus, results are presented for these methods only. Out of 21 species datasets, 3SEQ, Geneconv, RDP, and Bootscan detected signals in 20, 11, 11, and 7 datasets, respectively. Recombination was detected in all coronavirus species analyzed (6, 13, 1, and 1 species from the *Alpha*-, *Beta*-, *Delta-*, and *Gammacoronavirus*) by at least one method (Supplementary Table S1).

In total, 3SEQ found 443 recombination breakpoint pairs (RBPs), corresponding to 886 recombination breakpoints (RBs), 0.82% of the total RBPs identified (Table 1).

Coronavirus IBV (*Gammacoronavirus*) had the highest number of RBPs, namely, 137, and HKU2 (*Alphacoronavirus)* the lowest with 2 (Supplementary Table S2). Recombination breakpoints were often found at *ORF1b* (271 RBs) and rarely found at the *E* (7 RBs) gene (Table 1). It should be noted that RBs were calculated as the number of times one of the breakpoints fell in a given gene minus the number of times both of them occurred in the same gene.

Geneconv detected 579 RBPs (corresponding to 1158 RBs; 1.08%) with Coronavirus hCoV-NL63 (*Alphacoronavirus*) and TGEV (*Alphacoronavirus*) having the highest and lowest number of events at 222 and 2, respectively (Supplementary Table S2). *ORF1a* was the gene with the most RBs, 379, while *E* was the gene with the least RBs (8 RBs).

Bootscan registered a total of 345 RBPs (corresponding to 690 BPs; 0.64%), with Coronavirus TGEV being the species where most RBs were identified (111 RBs—Supplementary Table S2). The highest number of RBs was observed at the *Accessory* genes, with 313 RBs (Table 1).

The RDP approach produced the most RBPs, namely, 52,431 (corresponding to 104,862 RBs), 97.46% of the total (Table 1), and Coronavirus HKU15 (*Deltacoronavirus*) alone produced 37,506 RBPs (71.53% of the total; Supplementary Table S2). Most RBs were inferred to be located at *ORF1a* (as when using Geneconv), namely, 40,118, while the gene showing the least RBs was the *E* gene, with 488 (as when using Geneconv as well) (Table 2).

Despite the discrepancies in the number of RBs identified by Geneconv and RDP, across genes, the absolute (RB) results shown in Table 2 for these two methods are highly correlated (Spearman’s Rho = 1, *p* = 0; N = 7) and the correlation seems to be linear (Pearson’s R = 0.961; *p* < 0.001; N = 7). Research using these metrics can thus be directly compared after normalizing the results by the total number of recombination events that are detected using one of these two methods. The normalized results (%RB/%Alignment size) obtained with RDP were significantly correlated with those obtained when using both Geneconv and Bootscan (Spearman’s Rho = 0.857 and 0.786, respectively; *p* < 0.05; N = 7), and the correlation seemed to be linear, since RDP was significantly linearly correlated with Geneconv and Bootscan (Pearson’s R = 0.922 and 0.872, respectively; *p* < 0.01 and *p* < 0.05, respectively; N = 7). Moreover, Geneconv results were significantly linearly correlated with Bootscan results (Pearson’s R = 0.786; *p* < 0.05; N = 7); however, a non-parametric correlation was not statistically significant (Spearman’s Rho = 0.607, *p* > 0.05; N = 7). The results produced by 3SEQ were not significantly correlated with those produced by the other methods (0.143 < Spearman’s Rho < 0.286; *p* > 0.05; N = 7).

For the aggregated data (Table 1), across all four methods used, the average percentage of RBs for each genomic region was 37.91% for *ORF1a*, 23.03% for *ORF1b*, 10.75% for *S*, 0.47% for *E*, 1.73% for *M*, 8.59% for *N*, and 17.52% for *Accessory*.

Given the very different number of sequences available for different species, it was also important to determine its impact on RB detection. A significant linear correlation between dataset size (sequence/genome number) and number of inferred recombination events was found for RDP and Bootscan (Pearson’s R = 0.975 and 0.969, respectively; *p* < 0.001; N = 11 and 8). For 3SEQ and the dataset size, a significant correlation was also found (Spearman’s Rho = 0.712; *p* < 0.05; N = 21), although no significant linear correlation was found (Pearson’s R = 0.230, *p* > 0.05; N = 21). At the genomic region level, 3SEQ results presented statistically significant correlations with the dataset size for *ORF1a*, *ORF1b*, *S*, *M*, and *N* (0.561 < Spearman’s Rho < 0.667, *p* < 0.05; N = 21); however, no significant linear correlation was found (−0.185 < Pearson’s R < 0.336, *p* > 0.05; N = 21). Genevonv results presented no correlation with the dataset size (0.071 < Spearman’s Rho < 0.519, *p* > 0.05 and −0.308 < Pearson’s R < 0.488, *p* > 0.05; N = 12). When using RDP, linear correlations between dataset size and number of inferred recombination events were found for all genomic regions (0.779 < Spearman’s Rho < 0.936, *p* < 0.01 and 0.897 < Pearson’s R < 0.992, *p* < 0.001; N = 11). A statistically significant correlation between RB numbers and dataset size was also found when using Bootscan for all ORFs (0.801 < Spearman’s Rho < 0.866; *p* < 0.05; N = 8) with the exception of *E* and *Accessory* (Spearman’s Rho = 0.082 and 0.683, respectively; *p* > 0.05; N = 8). Furthermore, linear correlations were also found for Bootscan results with the dataset size, for all ORFs (0.768 < Pearson’s R < 0.956; *p* < 0.05; N = 8), with the exception of the *E* gene (Pearson’s R = 0.437; *p* > 0.05; N = 8).

Despite the sample size impact discussed above, which could introduce noise in gene recombination estimates for species with a low sample size, the gene recombination pattern observed for single species correlated well with that observed when using the aggregated data (Supplementary Table S1). For instance, when using Bootscan, for all six correlations that could be calculated (for SARS-CoV, a correlation could not be calculated because no RBs were detected in this species using Bootscan), Fisher’s R was above 0.80 (all correlations were statistically significant; *p* < 0.05). This suggests that all seven species (GCCDC1, HKU1, Murine-CoV, PHEV, SARS-CoV (*Betacoronavirus*), HKU2, and TGEV (*Alphacoronavirus*)) have similar recombination patterns. It should be noted that this method cannot be used when dealing with a large number of sequences, due to computational limitations. On the other hand, when using 3SEQ, which produced results for all species, although for SARS-CoV no RBs were detected, a statistically significant correlation was observed between gene recombination estimates in single species and that observed when using the aggregated data in 10 out of 20 cases (the Fisher’s R value was always above 0.75; Supplementary Table S1). With the exception of GCCDC1 (*Betacoronavirus*) and HKU10 (*Alphacoronavirus*), the sample size of the species showing a statistically significant correlation was always above 100. Therefore, it could be argued that species for which a high number of sequences was available contributed disproportionally to the aggregated estimates, and this could be the reason for the high Fisher’s R values observed when using such datasets. Nevertheless, the fact that a statistically significant correlation was observed for 10 species is still suggestive that there are no large changes in gene recombination patterns between different coronavirus species.

Gene order (*Accessory* genes were not considered due to their variable positioning) was significantly correlated with normalized recombination rate (%RB/%Alignment size) (Pearson’s R = 0.804; *p* < 0.05; N = 6). This pattern held only for the inference algorithms Geneconv and RDP (Pearson’s R = 0.987 and 0.801; *p* < 0.001 and 0.05, respectively). For 3SEQ and Bootscan, Pearson’s R was 0.002 and 0.217 with significance (*p*) of 0.455 and 0.340, respectively.

When looking at intratypic data, regarding the distribution of RBs and disregarding *E*/*E* due to its small size that prevented having two RBs in the same gene, the lowest nonzero observed/expected ratio was found at the *M* gene (0.34; Table 2), indicating that RBs were less prevalent at the *M* gene than expected under the hypothesis of an equal distribution of RBs along the genome, which is an approximation, given the above-mentioned correlation between gene order and normalized recombination rate. Nevertheless, given the position of this gene in the genome, the correlation between gene order and normalized recombination rate did not explain the low observed/expected ratio found at the *M* gene. The highest rate was observed for the *E*/*M* pair (7.91), and in that case, the correlation between gene order and normalized recombination rate may help explain this observation. For simplicity, the average gene boundaries were considered to be *ORF1a*–380–14,000; *ORF1b*–14,000–22,500; *S*–22,900–27,500; *E*–28,500–28,900; *M*–28,900–29,700; *N*–30,200–31,700. For *ORF1a* (with the exception of the *ORF1a*/*E* pair), *ORF1b*, *M*, and *N* genes (with the exception of the *ORF1b*/*N* pair), the observed/expected RB ratio was always lower for the cases where the two RBs fell within the same gene. This was in contrast to the *S* gene, supporting the view that this gene is often recombined as a unit (the cassette hypothesis) within species (Table 2).

When looking at intertypic data, all genes, with the exception of the *M* and *N* genes, seemed to be mainly recombined as a unit. For the *M* gene, most recombination events had an RB in the *M* gene and another at the *N* gene, as also observed when looking at intratypic data. Furthermore, contrary to intratypic data, the *E* gene, due to alignment gaps, now possessed enough size for some RBPs to be detected after our filtering.

### 2.2. Constrained vs. Unconstrained Same-Species Phylogenetic Trees

Recombination affecting one or a few genes only is predicted to produce incompatible gene trees when looking at different genes. Therefore, phylogenetic Bayesian trees were inferred using 21 same species datasets. In total, 147 unconstrained Bayesian trees were inferred, all of them achieving convergence (PSRF close to 1; Table 3). Moreover, 126 constrained Bayesian trees were inferred, with 122 achieving convergence (Table 3). The phylogenetic trees inferred using the *S* data were used for constraints acquisition. Convergence was not achieved for Coronavirus IBV (*ORF1a*), PHEV (*ORF1a* and whole genome), and SARS-related datasets (*M*). The large majority of constrained Bayesian trees were found to be incompatible with their unconstrained counterpart (*p* < 0.05). Indeed, constrained and unconstrained Bayesian trees were found to be compatible in just 18 comparisons involving the coronaviruses hCoV-HKU2 (for *ORF1b*, *E*, and *N*), TGEV (for *E*) (*Alphacoronavirus*), HKU1 (for *ORB1b*, *M*, and *N*), HKU4 (for *E* and *N*), HKU9 (for *E*), MERS-COV (animal hosts) (for *E*), MERS-CoV (human host) (for *E*), SARS-CoV (for *ORF1a*, *ORF1b*, *E*, *M*, and *N*), and SARS-CoV-2 (for *E*) (*Betacoronavirus*) datasets. In 8 out of 21 cases, the phylogenetic trees for *S* and *E* were found to be compatible (hCoV-HKU2, HKU4, HKU9, TGEV, MERS-CoV (animal hosts), MERS-CoV (human host), SARS-CoV, and SARS-CoV-2). Therefore, the phylogenetic analyses fully support the evidence for recombination.

### 2.3. Constrained vs. Unconstrained Different-Species Phylogenetic Trees

A similar approach to the one described above was used to identify intertypic recombination events. All unconstrained trees and the *E*, *M*, and *N* constrained trees achieved convergence (PSRF close to 1). All Bayesian constrained trees were less likely than their unconstrained counterparts (*p* < 0.05). It should be noted that the genome sequence selected from the MERS-CoV (animal hosts) dataset was identical to the one from the MERS-CoV-related dataset, hence, only a MERS-CoV-related (*Betacoronavirus*) sequence was used for the intertypic analysis. With the exception of the *M* and *E* genes, due to their relatively small size, all the others produced well-defined trees with high posterior credibility values (PCVs).

The unconstrained Bayesian trees show different evolutionary histories depending on the gene region being analyzed, with this phenomenon being observed at the genus level, showing how difficult it would be to classify these strains using information on a single gene. For instance, when looking at the whole genome, a split was observed between *Gamma*- and *Deltacoronavirus* and *Alpha*- and *Betacoronavirus* (Supplementary Figure S1). However, when we shifted the focus to *ORF1a*, the *Alphacoronaviruses* were the first group to split. The latter genus presented the most variation regarding inferred evolutionary histories. Indeed, it was the only genus that gave rise to more than one distinct cluster, two and four for both the *S* and *N* genes, respectively, in both cases, supported by high PCVs.

A clear example of an old intertypic recombination event between species from different genera, involving the *S* gene is shown in Supplementary Figure S1C, where *Alphacoronavirus* species belong to two different well-supported clusters, one of them clustering with *Betacoronavirus* and the second one with *Deltacoronavirus*.

*Alphacoronavirus* was the most inconsistent genus at the subgenera level. For instance, when looking at *ORF1a* and *ORF1b*, *Pedacovirus/Colacovirus* were the sister group of *Decacovirus* with high support. Nevertheless, they were not sister groups when looking at any of the other genes. Moreover, *Myotacovirus* and *Minunacovirus* were sister groups (with a high support value) when looking at the *S* gene only (Supplementary Figure S1).

Incongruencies were also observed in *Betacoronavirus* (Supplementary Figure S1). Namely, inferences based on the *S* gene showed that the sub-genera *Embecovirus* and *Merbecovirus* were sister groups, and this relationship was highly supported. Nevertheless, in all other trees, *Embecovirus* was never the sister group of *Merbecovirus.* For instance, when using sequences from the *N* gene, *Merbecovirus* was the sister group of *Sarbecovirus/Hibecovirus*, and this relationship is highly supported. Regarding *Gammacoronavirus*, when looking at the *S* gene, the *Igacovirus* sequences did not exclusively form a cluster, sharing it with the single sequence belonging to *Brangacovirus*, the sister group of *Igacovirus* of the same genus. These two cases were the only occurrences of a single subgenus not forming a cluster, supported by high PCVs.

At the species level, regarding *Alphacoronavirus*, the *Pedacovirus* PEDV (MF577027.1) sequence showed inconsistent highly supported relationships, for instance, at the *S* and *N* genes. The same is true for the Pipistrellus kuhlii-CoV 3398 sequence (*Nyctacovirus;* compare the results for the *S* and *N* genes, for instance). Regarding *Betacoronavirus*, only for *Embecovirus* were highly supported inconsistencies found (for instance, the sister group of China Rattus-CoV-HKU24 (KM349743.1) when looking at *ORF1a* and *ORF1b* was different but both were highly supported; the same was true for hCoV-OC43 (MW532119.1) when looking at the *ORF1a* and *N* genes, for instance). Regarding *Deltacoronavirus*, the position of the White eye-CoV-HKU16 (NC_016991.1; *Buldecovirus*) sequence was inconsistent but highly supported (compare, for instance, the results for the *ORF1a* and *S* genes). The same was true for the Munia-CoV-HKU13 (NC_011550.1) sequence (compare, for instance, the results for the *ORF1a* and *E* genes).

The discrepancies described above are compatible with cases of intertypic recombination. When they affect an entire subgenus, there is evidence for an ancient intertypic recombination event. Events affecting single species were uncovered for all genera.

### 2.4. Non-Synonymous Divergence (Ka)

When compared to the mean *Ka* between two species (calculated for the structural, *ORF1a* and *ORF1b* genes), a significant *Ka* difference (±2σ, where σ is the standard deviation) was observed for at least one gene when looking at 8 (hCoV-229E, HKU10, PEDV (*Alphacoronavirus*), GCCDC1, MERS-CoV (animal hosts), Murine-CoV, SARS-CoV-related (*Betacoronavirus*), and IBV (*Gammacoronavirus*)) out of the 21 species analyzed. These significant *Ka* differences were observed in at least one gene in comparisons involving a total of 134 genomes. Of these, 130 (97.01%) genomes showed a significant *Ka* difference at the *S* gene. These genomes were from the GCCDC1, HKU10, IBV, MERS-CoV (animal hosts), Murine-CoV, PEDV, and SARS-CoV-related datasets. In absolute numbers, GCCDC1 contributed the least (2 out of 9 genomes) and IBV the most (45 out of 45) genomes, while in percentage, hCoV-229 contributed the least (10.0%; 3 out of 30). Moreover, 13 genomes (9.70%: 13 out of 134) presented a significant *Ka* difference at the *E* gene (all 13 genomes were from PEDV). Moreover, 3 genomes also presented a significant *Ka* difference (2.24%; 3 out of 134) at the *N* gene (all from hCoV-229E).

If recombination is an important source of variability upon which selection can act, a correlation between *Ka* values and recombination estimates may be observed. A linear correlation was only found for Bootscan (Pearson’s R = 0.869; *p* < 0.05; N = 6) but significant correlations were found for Bootscan and RDP (Spearman’s Rho = 0.829 and 0.829, *p* < 0.05; N = 6).

When analyzing the intertypic datasets, 14 genomes showed a significant *Ka* difference in at least one gene. In this case, 12 (85.71%) genomes showed a significant *Ka* difference at *ORF1b*, but in contrast to expectations, the observed *Ka* value was always lower (<2σ, where σ is the standard deviation) than the mean of structural and *ORF1* genes. Two genomes showed a significantly increased *Ka* value at the *E* (7.14%) and *S* (7.14%) genes. A significant linear correlation was only found between *Ka* and Geneconv estimates for the intertypic data (Pearson’s R = −0.862; *p* < 0.05; N = 6); however, no statistically significant non-parametric correlations were found between them (Spearman’s Rho = −0.429, *p* > 0.05).

### 2.5. Recombination and Selection

**Figure 1:**
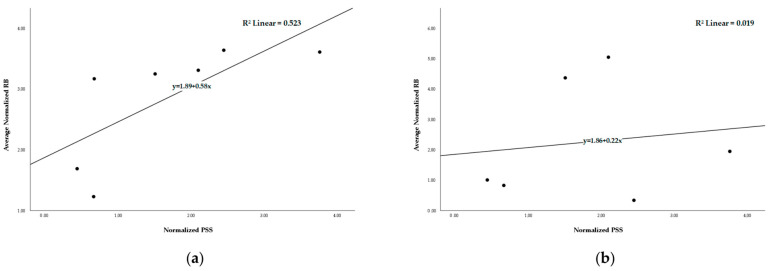
Graphical representation of the average normalized RBs obtained for intratypic (**a**) and intertypic (**b**) data and the corresponding normalized PSS values. The linear fit line with the corresponding equation and R^2^ is also shown.

Given the correlations that were observed between *Ka* values and recombination estimates, we also estimated the amount of variation (R^2^) that was explained under the hypothesis of a linear correlation between recombination rate and selection (measured as the ratio between Gene PSS/Total PSS and CDS Size/Total SARS-CoV-2 CDS, where PSS values were positively selected amino acid sites; estimates obtained from [35]). A statistically significant correlation was found between recombination and selection for intratypic data (Table 2; Spearman’s Rho = 0.929, *p* < 0.01); however, it was not linear (Pearson’s R = 0.723, *p* > 0.05) (Figure 1a).

In order to determine whether the significant correlation was the result of genes being recombined as a unit, as proposed for the *S* gene [19], we repeated the above analyses but this time using an estimate for the intragenic rate of recombination (number of cases where both RBs were found in the same gene divided by the gene length). No statistically significant correlation or linear correlation was found between recombination and selection (Spearman’s Rho = 0.086, *p* > 0.05; Pearson’s R = 0.138, *p* > 0.05) (Figure 1b). It should be noted that no intragenic rate of recombination could be estimated for the *E* gene, since the gene length was smaller than 500 bp, and in this work, RBPs smaller than 500 bp (see Section 4) were excluded. *Accessory* genes were treated as being a single large gene; hence, we must interpret such results with caution.

## 3. Discussion

Viruses generate genetic diversity through mutation, recombination, and in segmented RNA viruses (viruses that maintain their genomes as several distinct RNA molecules), through reassortment (the combination of genome segments from different parental origins) [1]. Such variability is essential for the adaptation to different hosts and efficient spreading. Here, we address the role of recombination, using multiple approaches (recombination inferences using RDP5, phylogenetic incongruency tests, and rates of non-synonymous evolution), in *Coronaviridae* and its putative role in species/strain adaptation. The use of multiple recombination inference methods (as in [26,36] and in this work) is important, since there are large discrepancies in the number of RBs identified by different methods, as we found here when using Bootscan (690 BPs) and RDP (104862 BPs), raising doubts on whether conclusions regarding recombination patterns could be influenced by the use of a given methodology. It should be noted, however, that despite large discrepancies in the number of inferred RBs, Geneconv (1158 BPs) and RDP (104862 BPs) results were highly linearly correlated across genes (Pearson’s R = 0.961; *p* < 0.001; N = 7). Therefore, if results are normalized by the total number of identified RBs, research using these metrics can be directly compared. Nevertheless, this is not always the case, since the results obtained with RDP are significantly correlated and significantly linearly correlated with those obtained with Geneconv and Bootscan (Spearman’s Rho = 0.857 and 0.786; *p* < 0.05; Pearson’s R = 0.922 and 0.872; *p* < 0.01 and *p* < 0.05, respectively; N = 7), but the results produced by 3SEQ were not significantly correlated with those produced by any of the other methods (0.143 < Spearman’s Rho < 0.286; *p > 0.05*; N = 7). This means that it is difficult to compare the results obtained by [8,33] (which only used 3SEQ) with those obtained by [31] (which only used Bootscan), for instance. Moreover, 3SEQ uses a matching method between a recombinant and its parental sequences (a triplet). Therefore, problems may arise when only one parental sequence is identified [40]. This is more likely to occur when analyzing smaller datasets, creating a bias that potentially leads to the observed lack of a correlation with the results obtained with other methods.

Correlations were found between the number of inferred RBs and sample size (number of genomes/sequences), and thus it may be difficult to compare recombination levels between species. Nevertheless, recombination estimates for single species were significantly correlated with the estimates obtained using data from all species, showing that the recombination landscape of different coronaviruses species is not very different.

The mixing of sequences from different subgenera is a common approach when a small number of genomes is available for each species, but this could in principle obscure intratypic recombination patterns if intertypic recombination patterns are very different, as shown here. At present, there are 21 species for which more than eight genome sequences are available (Table 1). Nevertheless, while *Alphacoronaviruses* and *Betacoronaviruses* are represented by six and thirteen species, respectively, *Deltacoronaviruses* and *Gammacoronaviruses* are represented by a single species. Because of the different sampling strategies, it is not possible to directly compare our results with those of [26,36] (which used all recombination inference methods but mixed sequences from different subgenera).

Intratypic recombination signals could be found in all species belonging to all four *Orthocoronavirinae* genera, when using all methods discussed here; however, recombination signals were not detected using 3SEQ in SARS-CoV, using Geneconv in PHEV and SARS-CoV, and using Bootscan in HKU4, PHEV, and SARS-CoV (*Betacoronavirus*). Moreover, intratypic recombination signals were found all across the genome, showing the importance of recombination in Coronaviruses evolution. Our results are different from those of [8,30], which mainly identified RB surrounding the *S* gene.

The gene order was found to be significantly positively correlated with the normalized recombination rate (Pearson’s R = 0.804; *p* < 0.05; N = 6), meaning that structural genes seem to experience more recombination than nonstructural genes. It should be noted that with the approach used here, we could only identify the recombination events that were not deleterious. *ORF1a* and *ORF1b* encode the machinery for virus replication [41]. Selective pressures may eliminate recombinant viruses, as changes in this region may lead to the loss of virus viability, partially explaining the observed correlation between gene order and normalized recombination rate.

Within species, only the *S* gene seemed to be recombined often as a unit. Given the high number of PSSs at the S protein (this protein recognizes the host’s receptor and is a primary target of the immune system [16]), it is conceivable that over time, within the S protein, incompatible amino acid variants will arise in different strains, meaning that recombinants involving only a fraction of the *S* gene would be deleterious. At the population level, this could be a minor issue for the other genes, leading to the observed lack of a correlation between intragenic recombination estimates and selection. This hypothesis has been previously advanced by others regarding recombination at the *S* gene [19,33,34], using genus level data. As expected under this hypothesis, given that most of the Coronavirus species here analyzed are very divergent, most intertypic recombination events are of the cassette type, independently of the gene considered, the exception being the *M* gene, for which most of the times, the RB pair falls in the *N* gene, a pattern that is also observed when looking at intratypic data. In coronaviruses, the N and M proteins have been shown to interact [42,43], and PSSs have been described in the interaction region of these two proteins [35]. Over time, incompatible M/N amino acid variant combinations may arise, explaining why the two genes are often recombined as one unit. The *N* gene has roles in regulating the virus life cycle, such as enabling the viral core formation and viral assembly. Furthermore, it modulates the host’s cell cycle and interferes with the host’s immune system [44,45,46], while the *M* gene also mediates viral assembly and budding in infected cells and has a role in inhibiting innate immune response by interfering with interferon production [47,48,49]. The results for the *Accessory* genes are more difficult to interpret because their relative location varies, and some genes may be species-specific.

The recombination inferences made here are supported by the phylogenetic analyses that showed incongruent evolutionary histories for different genes, as shown before by other authors, based on more restricted datasets [19,30,31,34,37]. Indeed, most (85.71%) phylogenetic trees were incompatible with the one inferred using data for the *S* gene (here used as reference). In the rare cases where similar evolutionary histories were found, most of the times, it was the evolutionary history of the *E* gene that was compatible with that of the *S* gene, as expected, given the small size of the *E* gene and the close proximity of the two genes (228 and 862 bps, respectively, in SARS-CoV-2). However, this is not always the explanation, as in HKU4 (*Alphacoronavirus*), hCoV-HKU1, HKU2, and SARS-CoV (*Betacoronavirus*), for instance, the *N* gene evolutionary history was compatible with the one inferred for the *S* gene. While for the last three species, the recombination levels were low, with HKU4 and SARS-CoV not producing results for at least one of the recombination detection algorithms, the same could not be said for hCoV-HKU1. As shown here, recombination influenced phylogenetic inferences at the genus, subgenus, and species levels. Therefore, phylogenetic analyses uncovered both old and more recent cases of intertypic recombination. Events affecting single species were uncovered for all genera.

Non-synonymous levels of variability were inflated, especially at the *S* gene, as expected if recombination was an important source of variability upon which selection could act. These findings were further supported by the positive correlation that was observed between PSS and recombination estimates.

In conclusion, the observed intra- and intertypic recombination patterns were shaped by selection, although it is conceivable that recombination varied along the genome of coronaviruses due to intrinsic changes in recombination rates, as well.

## 4. Materials and Methods

### 4.1. Sequence Retrieval

The *Coronaviridae* species list was obtained from the International Committee on Taxonomy of Viruses (ICTV—https://ictv.global/report/chapter/coronaviridae/taxonomy/coronaviridae accessed on 4 February 2024). Each species name was used as a query at NCBI Viruses (https://www.ncbi.nlm.nih.gov/labs/virus/vssi/#/; accessed on 10 February 2024). To avoid the presence of poorly annotated sequences, the Nucleotide Completeness and Ambiguous Characters built-in filters were selected. Therefore, only complete nucleotide sequences with a maximum of 300 ambiguous positions were retrieved. A single species was removed by the usage of these filters, namely, *Alphaletovirus microhylae*, the only representative of the *Letovirinae* subfamily. For SARS-CoV-2, more than two million sequences are available, and thus, only one representative of each strain was selected. In total, 7034 sequences from 58 species/datasets were retrieved. For recombination inferences, a list composed of 21 species (Table 4) was obtained after filtering for those species with more than eight genome sequences.

### 4.2. Recombination Inferences

For each of the 21 species listed in Table 1, the genomes were aligned using the Docker image for ClustalOmega tag 1.2.4 (http://bdip.i3s.up.pt/container/clustalomega, assessed on 9 February 2024 [50]), available at the pegi3s Bioinformatics Docker Images project (pegi3s-BDIP; [51], and ambiguous positions changed to “N”’s using Linux command line commands. The recombine (tag 1.0.0) Docker image, also available at the pegi3s-BDIP (http://bdip.i3s.up.pt/container/recombine, accessed on 12 February 2024), which facilitates the usage of the OpenRDP (RDP5) (https://western-bioinfo.github.io/software/OpenRDP assessed on 9 February 2024), 3SEQ [40], and Phipack [52] software applications, was used to infer recombination events. Given the sequence numbers’ heterogeneity, from as low as 9 for GCCDC1 to as high as 2794 for SARS-CoV-2, not all recombination detection methods could be used in every case. Species with as many as 150 sequences were analyzed with all methods present in OpenRDP (RDP5), i.e., 3SEQ, Geneconv, Bootscan, RDP, Chimaera, Siscan, and MaxChi. For the remaining species, due to computational limitations, only 3SEQ and Geneconv were used, or even just 3SEQ (in the case of SARS-CoV-2). SARS-CoV-2 sequences known to be recombinant (their PANGO designation starts with an “X”) were not used. Based on the RDP5 report, results were filtered to only include the ones with statistical support (*p* < 0.05). Only recombination fragments over 500 bp that did not fall in the first and/or last 1% of the genome alignment and that were smaller than 90% of the genome alignment were considered as true recombination events. In the analyses, when taking into account gene size, the value used was that of the average gene size based on the datasets used for a given method (i.e., 3SEQ, Geneconv, RDP, and Bootscan).

### 4.3. Phylogenetic Analysis

For intratypic phylogenetic analyses, the target number of selected sequences was around 30. Sequences identified as recombinant, when using multiple recombination inference methods, were given priority for inclusion in the dataset. From the genome alignment, used as input for RDP5 (see above), the start and stop codon of each gene in each genome of interest was identified, and the corresponding sequence isolated. This approach meant that we could use data from non-annotated genomes as well. The retrieved sequences were unaligned, translated, aligned at the amino acid level using ClustalOmega, and the corresponding nucleotide alignment obtained, using MEGAX (tag 10.0.5; a Docker image for this software application is available at pegi3s-BDIP (http://bdip.i3s.up.pt/container/megax, accessed on 21 February of 2024 [53]). New alignments for the whole genome were also obtained, this time using only the genomes of interest, using ClustalOmega as implemented in SEDA (tag 1.7.2; a Docker image for this software application is available at pegi3s-BDIP (http://bdip.i3s.up.pt/container/seda, accessed on 26 February 2024 [54]). Bayesian phylogenetic analyses were performed, using the MrBayes (tag 3.2.6) [55] Docker image available at the pegi3s-BDIP (http://bdip.i3s.up.pt/container/mrbayes, accessed on 8 March 2024). Two million generations, sampled every 100th generation, and a burn in of 2500 (25% of the samples) was used. Convergence was assessed using the potential scale reduction factor (PSRF) values. Therefore, for each species, seven phylogenetic trees were made, namely, one for *ORF1a*, *ORF1b*, *S*, *E*, *M*, *N*, and the whole genome. Tree visualization was carried out using MEGAX [53].

Sequence clusters with PCV above 89 were identified in the *S* Bayesian trees and imposed as constraints when inferring constrained trees for the other genes, using MrBayes and the same set of parameters as above. Thus, for each species, six new Bayesian trees were generated constrained on well-supported *S* sequence groups. To assess the compatibility of the unconstrained and constrained trees, the harmonic mean of both trees were compared using a Chi-Squared test. Tests with an associated *p* < 0.05 were considered significant.

In order to infer intertypic recombination events using a phylogenetic approach, the sequence with the longest branch length was selected from each of the genome datasets used for the intratypic analyses. We also added sequences from the species belonging to *Coronaviridae* that were not used for intratypic analyses due to their small sample size, using the same selection criteria, by performing a pairwise distance analysis in MEGAX [53]. In cases where only two sequences were available, the selection was random. Therefore, the final dataset was composed of 58 genome sequences. For all intertypic analyses, these 58 genomes were used as input. Then, seven FASTA files were created, as described above: ORF1a, ORF1b, S, E, M, N, and whole genome. These files were used as input for MrBayes for an identical phylogenetic analysis to the one described above.

All trees were rooted using the FastRoot [56] software (a Docker image (tag 1.5) is available at pegi3s-BDIP; http://bdip.i3s.up.pt/container/fastroot, accessed on 12 March 2024) using the Midpoint rooting method.

### 4.4. Non-Synonymous Rate (Ka) of Evolution

The *Ka* analysis was conducted on *ORF1a*, *ORF1b*, *S*, *E*, *M* and *N* genes. The codon alignments for each gene and species, used as input for MrBayes (see above), were used to estimate *Ka* values using kakscalculator ([57] Docker image (tag 2.0) available at the pegi3s -BDIP; http://bdip.i3s.up.pt/container/kakscalculator, accessed on 14 March 2024). Results were considered significant if the *Ka* estimate was at least two standard deviations above the average *Ka* for all genes.

### 4.5. Statistical Analyses

All statistical analyses were performed using SPSS (https://www.ibm.com/products/spss-statistics accessed on 14 March 2024, version 27.0.1.0).

## Figures and Tables

**Table 1:** Total number of RBs detected by RDP5’s methods for each gene, their corresponding percentage (%RB), and the ratio between %RB and %Alignment size.

Method		Gene							Total RB
		*ORF1a*	*ORF1b*	*S*	*E*	*M*	*N*	*Accessory*	
3SEQ	RB	174	271	220	7	22	34	158	886
	%RB	19.64	30.59	24.83	0.79	2.48	3.84	17.83	0.82
	%RB/%Alignment size	0.47	0.92	1.97	0.20	1.17	0.67	1.79	-
Geneconv	RB	379	314	129	8	41	50	237	1158
	%RB	32.73	27.12	11.14	0.69	3.54	4.32	20.47	1.08
	%RB/%Alignment size	0.58	0.82	1.07	1.34	1.36	1.67	2.25	-
RDP	RB	40118	24086	11094	488	1788	9148	18140	104862
	%RB	38.26	22.97	10.58	0.47	1.71	8.72	17.30	97.46
	%RB/%Alignment size	0.60	0.52	0.90	1.21	0.86	2.38	4.08	-
Bootscan	RB	116	105	123	7	12	14	313	690
	%RB	16.81	15.22	17.83	1.01	1.74	2.03	45.53	0.64
	%RB/%Alignment size	0.25	0.45	0.96	1.40	0.45	0.51	5.40	-
Total per gene	RB	40787	24776	11566	510	1863	9246	18848	107596
	%RB	37.91	23.03	10.75	0.47	1.73	8.59	17.52	-
	%RB/%Alignment size	0.47	0.68	1.23	1.04	0.96	1.31	3.38	-

**Table 2:** Ratio of the observed percentage of recombination breakpoint pairs per the expected percentage of recombination breakpoints given a random distribution. The first column represents the location of the first breakpoint and the second column the location of the second breakpoint. NA—not available.

Genomic Region		Intratypic		Intertypic	
		Observed/Expected	Average	Observed/Expected	Average
*ORF1a*	*ORF1a*	0.83	1.23	2.04	0.59
	*ORF1b*	2.02		1.35	
	*S*	1.25		0.59	
	*E*	0.64		0.00	
	*M*	0.97		0.00	
	*N*	2.03		0.00	
	*Accessory*	0.83		0.12	
*ORF1b*	*ORF1b*	1.01	1.69	13.19	2.25
	*S*	2.49		0.22	
	*E*	1.60		0.00	
	*M*	1.18		0.00	
	*N*	1.22		0.00	
	*Accessory*	2.28		1.00	
*S*	*S*	5.05	3.31	8.07	1.70
	*E*	2.37		0.00	
	*M*	4.72		0.00	
	*N*	3.39		0.54	
	*Accessory*	3.94		2.50	
*E*	*E*	NA	3.17	16.73	2.43
	*M*	7.91		1.95	
	*N*	6.18		0.00	
	*Accessory*	3.50		0.73	
*M*	*M*	0.34	3.64	2.38	2.04
	*N*	6.51		9.75	
	*Accessory*	3.86		0.17	
*N*	*N*	1.95	3.61	3.80	2.03
	*Accessory*	3.98		0.15	
*Accessory*	*Accessory*	4.37	3.25	0.17	0.69

**Table 3:** Distribution of constrained and unconstrained Bayesian trees, separated by their respective genomic region, followed by their respective number of convergent phylogenetic trees.

**Gene**	**Statistically Significant Trees**	**Statistically Insignificant Trees**	**# of Converged Trees**	**Total**
*ORF1a*	20 (95.24%)	1 (4.76%)	19 (85.71%)	21
*ORF1b*	18 (85.71%)	3 (14.29%)	21 (100%)	
*E*	13 (61.90%)	8 (38,10%)	21 (100%)	
*M*	19 (90.48%)	2 (9.52%)	20 (95.24%)	
*N*	17 (80.95%)	4 (19.05%)	21 (100%)	
Whole Genome	21 (100%)	0 (0%)	20 (95.24%)	
Total	108 (85.71%)	18 (14.29%)	122 (96.83%)	126

**Table 4:** List of datasets, their respective genus, and the number of sequences used as input for RDP5.

Dataset	Genus	Number of Sequences
hCoV-229E	*Alphacoronavirus*	152
HKU2	*Alphacoronavirus*	10
HKU10	*Alphacoronavirus*	25
hCoV-NL63	*Alphacoronavirus*	151
PEDV	*Alphacoronavirus*	851
TGEV	*Alphacoronavirus*	62
GCCDC1	*Betacoronavirus*	9
hCoV-HKU1	*Betacoronavirus*	63
HKU4	*Betacoronavirus*	10
HKU9	*Betacoronavirus*	10
MERS-CoV (animal hosts)	*Betacoronavirus*	334
MERS-CoV (human host)	*Betacoronavirus*	286
MERS-CoV-related	*Betacoronavirus*	631
Murine-CoV	*Betacoronavirus*	38
hCoV-OC43	*Betacoronavirus*	342
PHEV	*Betacoronavirus*	18
SARS-CoV	*Betacoronavirus*	13
SARS-CoV-2	*Betacoronavirus*	2794
SARS-CoV-related	*Betacoronavirus*	276
HKU15	*Deltacoronavirus*	200
IBV	*Gammacoronavirus*	667
37 species with fewer than eight sequences	-	92
Total	-	7034

## Data Availability

Data contained within the article.

## Supplementary Materials

The following supporting information can be downloaded at: https://www.mdpi.com/article/10.3390/ijms26125595/s1.
