# Supplementary material for: Patterns of Recombination in Coronaviruses
Source: Int J Mol Sci. 2025 Jun 11;26(12):5595. doi: 10.3390/ijms26125595 (PMC12193178; doi:10.3390/ijms26125595)
Supplement: Supplementary file 1 [file ijms-26-05595-s001.zip › Supplementary Figure 1.pdf]

A

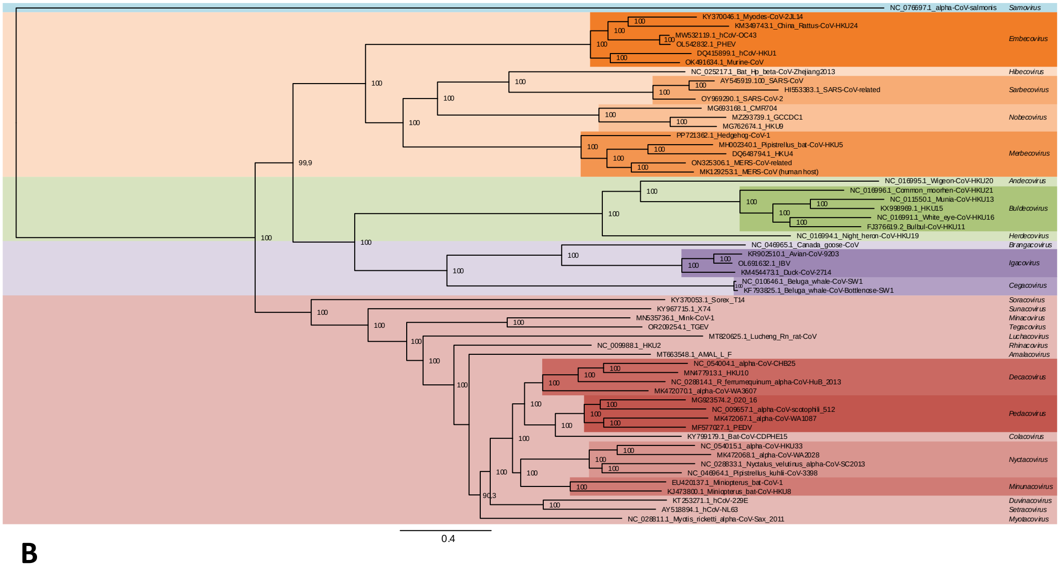
NC_076697.1_alpha-CoV-salmonis
Samovirus
KY370046.1_Myodes-CoV-2JL14
KM349743.1_China_Rattus-CoV-HKU24
MW532119.1_hCoV-OC43
OL542832.1_PHEV
DQ415899.1_hCoV-HKU1
OK491634.1_Murine-CoV
Embecovirus
NC_025217.1_Bat_Hp_beta-CoV-Zhejiang2013
Hibecovirus
AY545919.1_SARS-CoV
HI553383.1_SARS-CoV-related
OY969290.1_SARS-CoV-2
Sarbecovirus
MG693168.1_CMR704
MZ293739.1_GCCDC1
MG762674.1_HKU9
Nobecovirus
PP721362.1_Hedgehog-CoV-1
MH002340.1_Pipistrellus_bat-CoV-HKU5
DQ648794.1_HKU4
ON325306.1_MERS-CoV-related
MK129253.1_MERS-CoV (human host)
Merbecovirus
NC_016995.1_Wigeon-CoV-HKU20
Andecovirus
NC_016996.1_Common_moorhen-CoV-HKU21
NC_011550.1_Munia-CoV-HKU13
KX998969.1_HKU15
NC_016991.1_White_eye-CoV-HKU16
FJ376619.2_Bulbul-CoV-HKU11
Buldecovirus
NC_016994.1_Night_heron-CoV-HKU19
Herdecovirus
NC_046965.1_Canada_goose-CoV
Brangacovirus
KR902510.1_Avian-CoV-9203
OL691632.1_IBV
KM454473.1_Duck-CoV-2714
Igacovirus
NC_010646.1_Beluga_whale-CoV-SW1
KF793825.1_Beluga_whale-CoV-Bottlenose-SW1
Cegacovirus
KY370053.1_Sorex_T14
Soracovirus
KY967715.1_X74
Sunacovirus
MN535736.1_Mink-CoV-1
Minacovirus
OR209254.1_TGEV
Tegacovirus
MT820625.1_Lucheng_Rn_rat-CoV
Luchacovirus
NC_009988.1_HKU2
Rhinacovirus
MT663548.1_AMAL_L_F
Amalacovirus
NC_054004.1_alpha-CoV-CHB25
MN477913.1_HKU10
NC_028814.1_R_ferrumequinum_alpha-CoV-HuB_2013
MK472070.1_alpha-CoV-WA3607
Decacovirus
MG923574.2_020_16
NC_009657.1_alpha-CoV-scotophili_512
MK472067.1_alpha-CoV-WA1087
MF577027.1_PEDV
Pedacovirus
KY799179.1_Bat-CoV-CDPHE15
Colacovirus
NC_054015.1_alpha-CoV-HKU33
MK472068.1_alpha-CoV-WA2028
NC_028833.1_Nyctalus_velutinus_alpha-CoV-SC2013
NC_046964.1_Pipistrellus_kuhlii-CoV-3398
Nyctacovirus
EU420137.1_Miniopterus_bat-CoV-1
KJ473800.1_Miniopterus_bat-CoV-HKU8
Minunacovirus
KT253271.1_hCoV-229E
Duvinacovirus
AY518894.1_hCoV-NL63
Setracovirus
NC_028811.1_Myotis_ricketti_alpha-CoV-Sax_2011
Myotacovirus
0.4

B

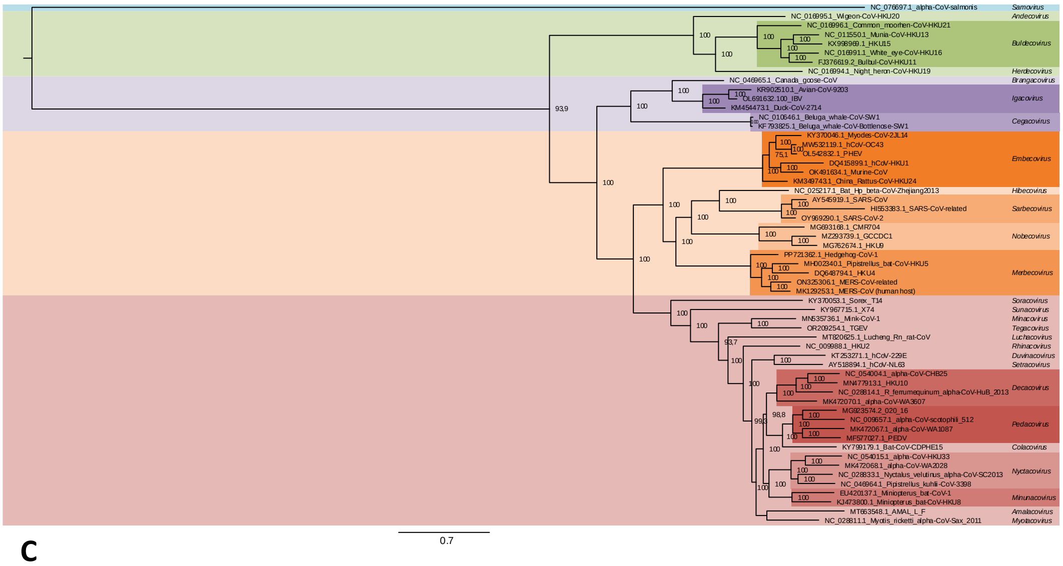
NC_076697.1_alpha-CoV-salmonis
Samovirus
NC_016995.1_Wigeon-CoV-HKU20
Andecovirus
NC_016996.1_Common_moorhen-CoV-HKU21
NC_011550.1_Munia-CoV-HKU13
KX998969.1_HKU15
NC_016991.1_White_eye-CoV-HKU16
FJ376619.2_Bulbul-CoV-HKU11
Buldecovirus
NC_016994.1_Night_heron-CoV-HKU19
Herdecovirus
NC_046965.1_Canada_goose-CoV
Brangacovirus
KR902510.1_Avian-CoV-9203
OL691632.1_IBV
KM454473.1_Duck-CoV-2714
Igacovirus
NC_010646.1_Beluga_whale-CoV-SW1
KF793825.1_Beluga_whale-CoV-Bottlenose-SW1
Cegacovirus
KY370046.1_Myodes-CoV-2JL14
MW532119.1_hCoV-OC43
OL542832.1_PHEV
DQ415899.1_hCoV-HKU1
OK491634.1_Murine-CoV
KM349743.1_China_Rattus-CoV-HKU24
Embecovirus
NC_025217.1_Bat_Hp_beta-CoV-Zhejiang2013
Hibecovirus
AY545919.1_SARS-CoV
HI553383.1_SARS-CoV-related
OY969290.1_SARS-CoV-2
Sarbecovirus
MG693168.1_CMR704
MZ293739.1_GCCDC1
MG762674.1_HKU9
Nobecovirus
PP721362.1_Hedgehog-CoV-1
MH002340.1_Pipistrellus_bat-CoV-HKU5
DQ648794.1_HKU4
ON325306.1_MERS-CoV-related
MK129253.1_MERS-CoV (human host)
Merbecovirus
KY370053.1_Sorex_T14
Soracovirus
KY967715.1_X74
Sunacovirus
MN535736.1_Mink-CoV-1
Minacovirus
OR209254.1_TGEV
Tegacovirus
MT820625.1_Lucheng_Rn_rat-CoV
Luchacovirus
NC_009988.1_HKU2
Rhinacovirus
KT253271.1_hCoV-229E
Duvinacovirus
AY518894.1_hCoV-NL63
Setracovirus
NC_054004.1_alpha-CoV-CHB25
MN477913.1_HKU10
NC_028814.1_R_ferrumequinum_alpha-CoV-HuB_2013
MK472070.1_alpha-CoV-WA3607
Decacovirus
MG923574.2_020_16
NC_009657.1_alpha-CoV-scotophili_512
MK472067.1_alpha-CoV-WA1087
MF577027.1_PEDV
Pedacovirus
KY799179.1_Bat-CoV-CDPHE15
Colacovirus
NC_054015.1_alpha-CoV-HKU33
MK472068.1_alpha-CoV-WA2028
NC_028833.1_Nyctalus_velutinus_alpha-CoV-SC2013
NC_046964.1_Pipistrellus_kuhlii-CoV-3398
Nyctacovirus
EU420137.1_Miniopterus_bat-CoV-1
KJ473800.1_Miniopterus_bat-CoV-HKU8
Minunacovirus
MT663548.1_AMAL_L_F
Amalacovirus
NC_028811.1_Myotis_ricketti_alpha-CoV-Sax_2011
Myotacovirus
0.7

C

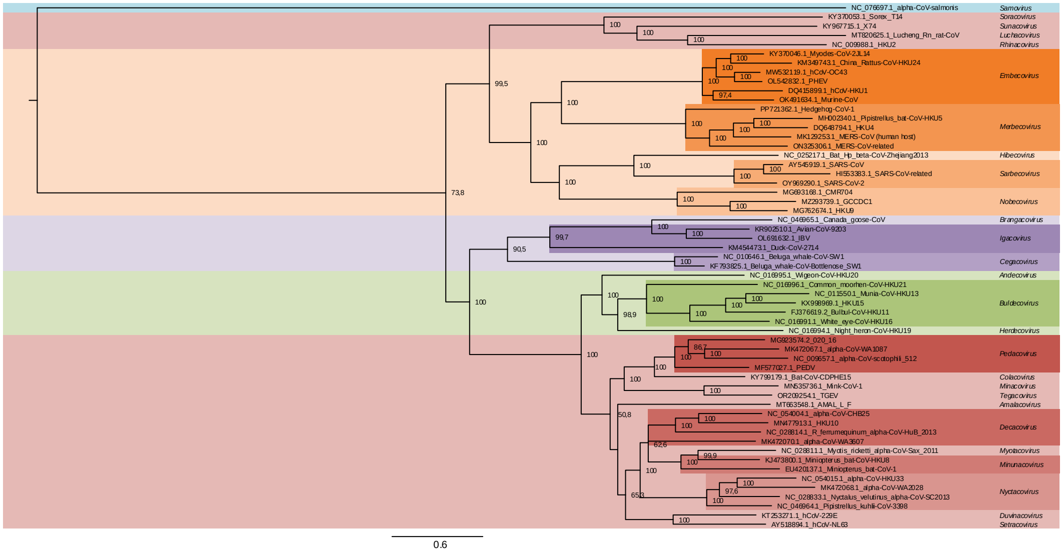
NC_076697.1_alpha-CoV-salmonis
Samovirus
KY370053.1_Sorex_T14
Soracovirus
KY967715.1_X74
Sunacovirus
MT820625.1_Lucheng_Rn_rat-CoV
Luchacovirus
NC_009988.1_HKU2
Rhinacovirus
KY370046.1_Myodes-CoV-2JL14
KM349743.1_China_Rattus-CoV-HKU24
MW532119.1_hCoV-OC43
OL542832.1_PHEV
DQ415899.1_hCoV-HKU1
OK491634.1_Murine-CoV
Embecovirus
PP721362.1_Hedgehog-CoV-1
MH002340.1_Pipistrellus_bat-CoV-HKU5
DQ648794.1_HKU4
MK129253.1_MERS-CoV (human host)
ON325306.1_MERS-CoV-related
Merbecovirus
NC_025217.1_Bat_Hp_beta-CoV-Zhejiang2013
Hibecovirus
AY545919.1_SARS-CoV
HI553383.1_SARS-CoV-related
OY969290.1_SARS-CoV-2
Sarbecovirus
MG693168.1_CMR704
MZ293739.1_GCCDC1
MG762674.1_HKU9
Nobecovirus
NC_046965.1_Canada_goose-CoV
Brangacovirus
KR902510.1_Avian-CoV-9203
OL691632.1_IBV
KM454473.1_Duck-CoV-2714
Igacovirus
NC_010646.1_Beluga_whale-CoV-SW1
KF793825.1_Beluga_whale-CoV-Bottlenose_SW1
Cegacovirus
NC_016995.1_Wigeon-CoV-HKU20
Andecovirus
NC_016996.1_Common_moorhen-CoV-HKU21
NC_011550.1_Munia-CoV-HKU13
KX998969.1_HKU15
FJ376619.2_Bulbul-CoV-HKU11
NC_016991.1_White_eye-CoV-HKU16
Buldecovirus
NC_016994.1_Night_heron-CoV-HKU19
Herdecovirus
MG923574.2_020_16
MK472067.1_alpha-CoV-WA1087
NC_009657.1_alpha-CoV-scotophili_512
MF577027.1_PEDV
Pedacovirus
KY799179.1_Bat-CoV-CDPHE15
Colacovirus
MN535736.1_Mink-CoV-1
Minacovirus
OR209254.1_TGEV
Tegacovirus
MT663548.1_AMAL_L_F
Amalacovirus
NC_054004.1_alpha-CoV-CHB25
MN477913.1_HKU10
NC_028814.1_R_ferrumequinum_alpha-CoV-HuB_2013
MK472070.1_alpha-CoV-WA3607
Decacovirus
NC_028811.1_Myotis_ricketti_alpha-CoV-Sax_2011
Myotacovirus
KJ473800.1_Miniopterus_bat-CoV-HKU8
EU420137.1_Miniopterus_bat-CoV-1
Minunacovirus
NC_054015.1_alpha-CoV-HKU33
MK472068.1_alpha-CoV-WA2028
NC_028833.1_Nyctalus_velutinus_alpha-CoV-SC2013
NC_046964.1_Pipistrellus_kuhlii-CoV-3398
Nyctacovirus
KT253271.1_hCoV-229E
Duvinacovirus
AY518894.1_hCoV-NL63
Setracovirus
0.6

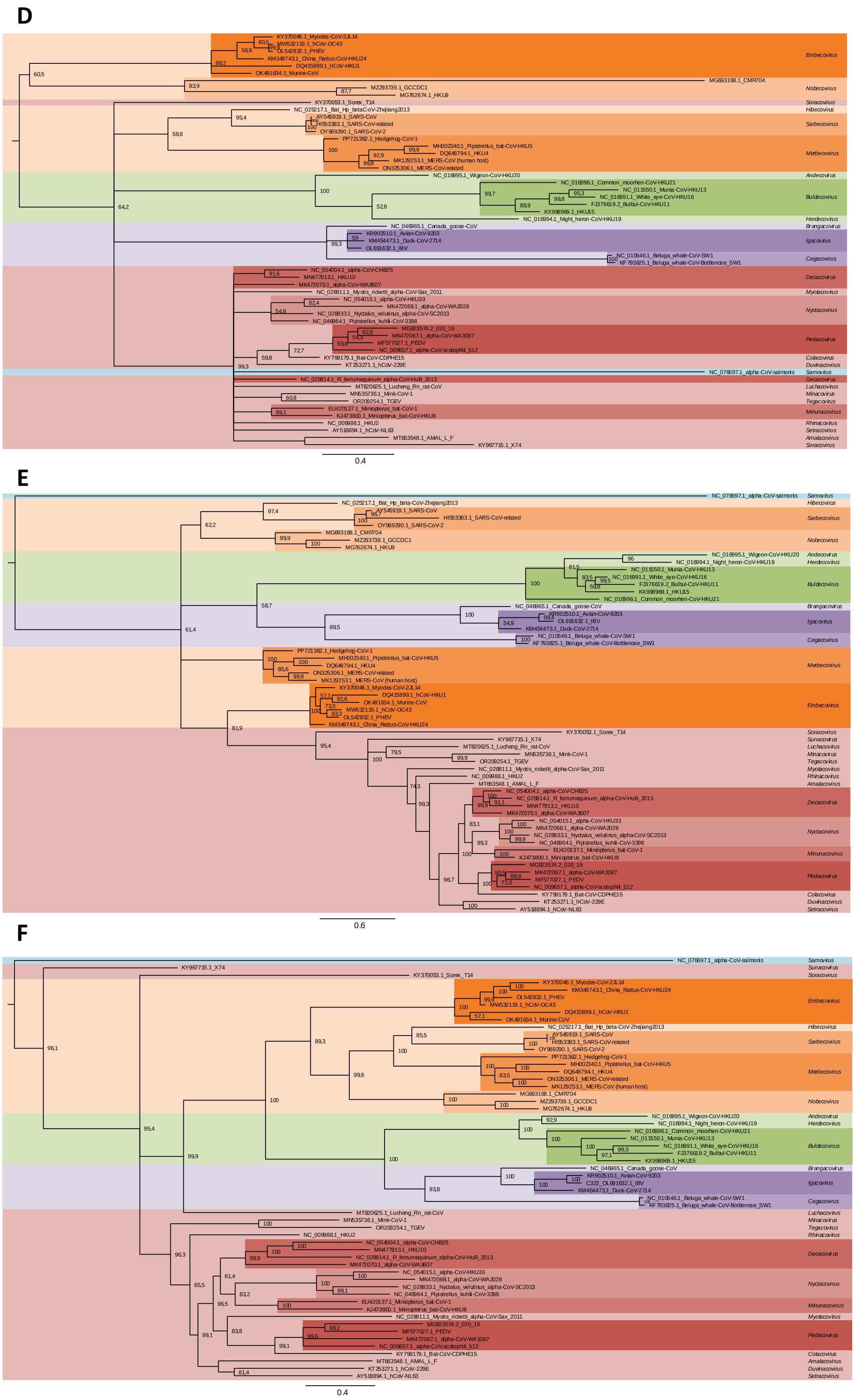
D
KY370046.1_Myodes-CoV-2JL14
MW532119.1_hCoV-OC43
OL542832.1_PHEV
KM349743.1_China_Rattus-CoV-HKU24
DQ415899.1_hCoV-HKU1
OK491634.1_Murine-CoV
Embecovirus
MG693168.1_CMR704
MZ293739.1_GCCDC1
MG762674.1_HKU9
Nobecovirus
KY370053.1_Sorex_T14
Soracovirus
NC_025217.1_Bat_Hp_betaCoV-Zhejiang2013
Hibecovirus
AY545919.1_SARS-CoV
HI553383.1_SARS-CoV-related
OY969290.1_SARS-CoV-2
Sarbecovirus
PP721362.1_Hedgehog-CoV-1
MH002340.1_Pipistrellus_bat-CoV-HKU5
DQ648794.1_HKU4
MK129253.1_MERS-CoV (human host)
ON325306.1_MERS-CoV-related
Merbecovirus
NC_016995.1_Wigeon-CoV-HKU20
Andecovirus
NC_016996.1_Common_moorhen-CoV-HKU21
NC_011550.1_Munia-CoV-HKU13
NC_016991.1_White_eye-CoV-HKU16
FJ376619.2_Bulbul-CoV-HKU11
KX998969.1_HKU15
Buldecovirus
NC_016994.1_Night_heron-CoV-HKU19
Herdecovirus
NC_046965.1_Canada_goose-CoV
Brangacovirus
KR902510.1_Avian-CoV-9203
KM454473.1_Duck-CoV-2714
OL691632.1_IBV
Igacovirus
NC_010646.1_Beluga_whale-CoV-SW1
KF793825.1_Beluga_whale-CoV-Bottlenose_SW1
Cegacovirus
NC_054004.1_alpha-CoV-CHB25
MN477913.1_HKU10
MK472070.1_alpha-CoV-WA3607
Decacovirus
NC_028811.1_Myotis_ricketti_alpha-CoV-Sax_2011
Myotacovirus
NC_054015.1_alpha-CoV-HKU33
MK472068.1_alpha-CoV-WA2028
NC_028833.1_Nyctalus_velutinus_alpha-CoV-SC2013
NC_046964.1_Pipistrellus_kuhlii-CoV-3398
Nyctacovirus
MG923574.2_020_16
MK472067.1_alpha-CoV-WA1087
MF577027.1_PEDV
NC_009657.1_alpha-CoV-scotophili_512
Pedacovirus
KY799179.1_Bat-CoV-CDPHE15
Colacovirus
KT253271.1_hCoV-229E
Duvinacovirus
NC_076697.1_alpha-CoV-salmonis
Samovirus
NC_028814.1_R_ferrumequinum_alpha-CoV-HuB_2013
Decacovirus
MT820625.1_Lucheng_Rn_rat-CoV
Luchacovirus
MN535736.1_Mink-CoV-1
Minacovirus
OR209254.1_TGEV
Tegacovirus
EU420137.1_Miniopterus_bat-CoV-1
KJ473800.1_Miniopterus_bat-CoV-HKU8
Minunacovirus
NC_009988.1_HKU2
Rhinacovirus
AY518894.1_hCoV-NL63
Setracovirus
MT663548.1_AMAL_L_F
Amalacovirus
KY967715.1_X74
Soracovirus
0.4
E
NC_076697.1_alpha-CoV-salmonis
Samovirus
NC_025217.1_Bat_Hp_beta-CoV-Zhejiang2013
Hibecovirus
AY545919.1_SARS-CoV
HI553383.1_SARS-CoV-related
OY969290.1_SARS-CoV-2
Sarbecovirus
MG693168.1_CMR704
MZ293739.1_GCCDC1
MG762674.1_HKU9
Nobecovirus
NC_016995.1_Wigeon-CoV-HKU20
Andecovirus
NC_016994.1_Night_heron-CoV-HKU19
Herdecovirus
NC_011550.1_Munia-CoV-HKU13
NC_016991.1_White_eye-CoV-HKU16
FJ376619.2_Bulbul-CoV-HKU11
KX998969.1_HKU15
NC_016996.1_Common_moorhen-CoV-HKU21
Buldecovirus
NC_046965.1_Canada_goose-CoV
Brangacovirus
KR902510.1_Avian-CoV-9203
OL691632.1_IBV
KM454473.1_Duck-CoV-2714
Igacovirus
NC_010646.1_Beluga_whale-CoV-SW1
KF793825.1_Beluga_whale-CoV-Bottlenose_SW1
Cegacovirus
PP721362.1_Hedgehog-CoV-1
MH002340.1_Pipistrellus_bat-CoV-HKU5
DQ648794.1_HKU4
ON325306.1_MERS-CoV-related
MK129253.1_MERS-CoV (human host)
Merbecovirus
KY370046.1_Myodes-CoV-2JL14
DQ415899.1_hCoV-HKU1
OK491634.1_Murine-CoV
MW532119.1_hCoV-OC43
OL542832.1_PHEV
KM349743.1_China_Rattus-CoV-HKU24
Embecovirus
KY370053.1_Sorex_T14
Soracovirus
KY967715.1_X74
Sunacovirus
MT820625.1_Lucheng_Rn_rat-CoV
Luchacovirus
MN535736.1_Mink-CoV-1
Minacovirus
OR209254.1_TGEV
Tegacovirus
NC_028811.1_Myotis_ricketti_alpha-CoV-Sax_2011
Myotacovirus
NC_009988.1_HKU2
Rhinacovirus
MT663548.1_AMAL_L_F
Amalacovirus
NC_054004.1_alpha-CoV-CHB25
NC_028814.1_R_ferrumequinum_alpha-CoV-HuB_2013
MN477913.1_HKU10
MK472070.1_alpha-CoV-WA3607
Decacovirus
NC_054015.1_alpha-CoV-HKU33
MK472068.1_alpha-CoV-WA2028
NC_028833.1_Nyctalus_velutinus_alpha-CoV-SC2013
NC_046964.1_Pipistrellus_kuhlii-CoV-3398
Nyctacovirus
EU420137.1_Miniopterus_bat-CoV-1
KJ473800.1_Miniopterus_bat-CoV-HKU8
Minunacovirus
MG923574.2_020_16
MK472067.1_alpha-CoV-WA1087
MF577027.1_PEDV
NC_009657.1_alpha-CoV-scotophili_512
Pedacovirus
KY799179.1_Bat-CoV-CDPHE15
Colacovirus
KT253271.1_hCoV-229E
Duvinacovirus
AY518894.1_hCoV-NL63
Setracovirus
0.6
F
NC_076697.1_alpha-CoV-salmonis
Samovirus
KY967715.1_X74
Sunacovirus
KY370053.1_Sorex_T14
Soracovirus
KY370046.1_Myodes-CoV-2JL14
KM349743.1_China_Rattus-CoV-HKU24
OL542832.1_PHEV
MW532119.1_hCoV-OC43
DQ415899.1_hCoV-HKU1
OK491634.1_Murine-CoV
Embecovirus
NC_025217.1_Bat_Hp_beta-CoV-Zhejiang2013
Hibecovirus
AY545919.1_SARS-CoV
HI553383.1_SARS-CoV-related
OY969290.1_SARS-CoV-2
Sarbecovirus
PP721362.1_Hedgehog-CoV-1
MH002340.1_Pipistrellus_bat-CoV-HKU5
DQ648794.1_HKU4
ON325306.1_MERS-CoV-related
MK129253.1_MERS-CoV (human host)
Merbecovirus
MG693168.1_CMR704
MZ293739.1_GCCDC1
MG762674.1_HKU9
Nobecovirus
NC_016995.1_Wigeon-CoV-HKU20
Andecovirus
NC_016994.1_Night_heron-CoV-HKU19
Herdecovirus
NC_016996.1_Common_moorhen-CoV-HKU21
NC_011550.1_Munia-CoV-HKU13
NC_016991.1_White_eye-CoV-HKU16
FJ376619.2_Bulbul-CoV-HKU11
KX998969.1_HKU15
Buldecovirus
NC_046965.1_Canada_goose-CoV
Brangacovirus
KR902510.1_Avian-CoV-9203
C322_OL691632.1_IBV
KM454473.1_Duck-CoV-2714
Igacovirus
NC_010646.1_Beluga_whale-CoV-SW1
KF793825.1_Beluga_whale-CoV-Bottlenose_SW1
Cegacovirus
MT820625.1_Lucheng_Rn_rat-CoV
Luchacovirus
MN535736.1_Mink-CoV-1
Minacovirus
OR209254.1_TGEV
Tegacovirus
NC_009988.1_HKU2
Rhinacovirus
NC_054004.1_alpha-CoV-CHB25
MN477913.1_HKU10
NC_028814.1_R_ferrumequinum_alpha-CoV-HuB_2013
MK472070.1_alpha-CoV-WA3607
Decacovirus
NC_054015.1_alpha-CoV-HKU33
MK472068.1_alpha-CoV-WA2028
NC_028833.1_Nyctalus_velutinus_alpha-CoV-SC2013
NC_046964.1_Pipistrellus_kuhlii-CoV-3398
Nyctacovirus
EU420137.1_Miniopterus_bat-CoV-1
KJ473800.1_Miniopterus_bat-CoV-HKU8
Minunacovirus
NC_028811.1_Myotis_ricketti_alpha-CoV-Sax_2011
Myotacovirus
MG923574.2_020_16
MF577027.1_PEDV
MK472067.1_alpha-CoV-WA1087
NC_009657.1_alpha-CoV-scotophili_512
Pedacovirus
KY799179.1_Bat-CoV-CDPHE15
Colacovirus
MT663548.1_AMAL_L_F
Amalacovirus
KT253271.1_hCoV-229E
Duvinacovirus
AY518894.1_hCoV-NL63
Setracovirus
0.4

G

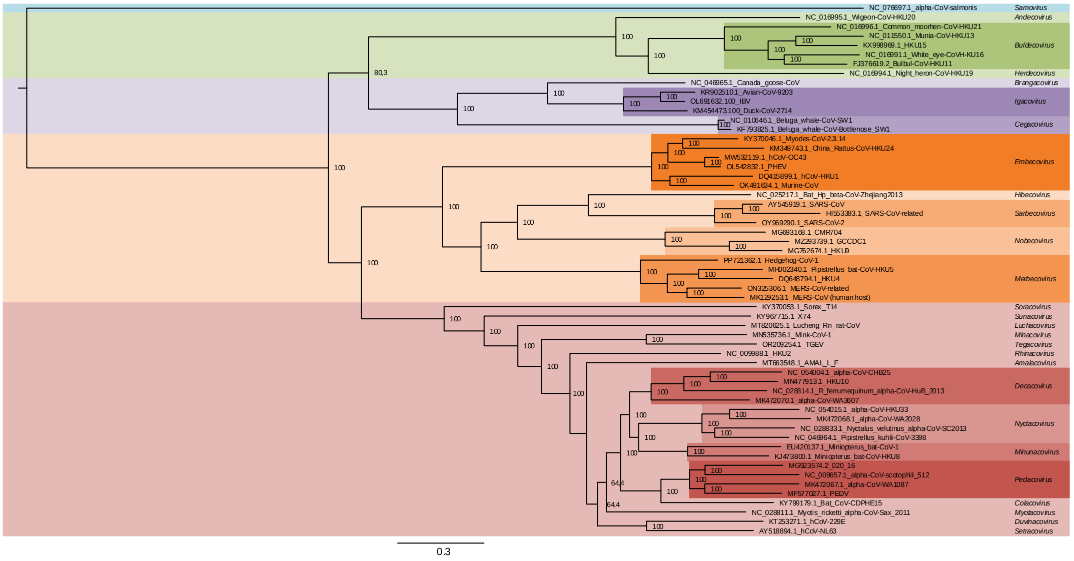

Supplementary Figure 1 – Bayesian trees of the intertypic data, produced by MrBayes with two million generations with a burnin of 2500. Background colors represent genera, with different shading subgenera (named on the right). Red – *Alphacoronavirus*, orange – *Betacoronavirus*, green – *Deltacoronavirus*, purple – *Gammacoronavirus* and blue – *Alphapironavirus*. (A) ORF1a, (B) ORF1b, (C) Spike, (D) Envelope, (E) Membrane, (F) Nucleocapsid and (G) Whole Genome. Numbers represent the posterior credibility value.
