# Supplementary material for: Patterns of Recombination in Coronaviruses
Source: Int J Mol Sci. 2025 Jun 11;26(12):5595. doi: 10.3390/ijms26125595 (PMC12193178; doi:10.3390/ijms26125595)
Supplement: Supplementary file 1 [file ijms-26-05595-s001.zip › Supplementary tables 1 and 2.pdf]

Supplementary Table 1. Correlation coefficient values (Spearman's Rho and Pearson's R) obtained when comparing the recombination breakpoint distribution of a single species with the one of the Aggregate (Supplementary Table 1). **P*<0.05; ***P*<0.01; N = 7.

Genus	Species	Correlation test	3seq	Geneconv	RDP	Bootscan
Alphacoronavirus	hCoV-229E	Spearman's Rho	0,617	0,748	-	-
		Pearson's R	0,693	0,717	-	-
	hCoV-NL63	Spearman's Rho	0,927**	0,927**	-	-
		Pearson's R	0,938**	0,849*	-	-
	HKU10	Spearman's Rho	0,991**	0,429	0,893**	-
		Pearson's R	0,940**	0,321	0,567	-
	HKU2	Spearman's Rho	0,478	0,873*	0,556	0,808*
		Pearson's R	0,532	0,905**	0,536	0,887**
	PEDV	Spearman's Rho	0,964**	-	-	-
		Pearson's R	0,922**	-	-	-
	TGEV	Spearman's Rho	0,655	0,598	0,893**	1**
		Pearson's R	0,702	0,559	0,492	0,951**
	GCCDC1	Spearman's Rho	0,778*	0,532	0,18	0,43
		Pearson's R	0,785*	0,346	0,15	0,896**
Betacoronavirus	hCoV-HKU1	Spearman's Rho	0,632	0,883**	0,679	0,847*
		Pearson's R	0,621	0,721	0,832*	0,869*
	hCoV-OC43	Spearman's Rho	0,655	-	-	-
		Pearson's R	0,617	-	-	-
	HKU4	Spearman's Rho	0,579	0,815*	0,746	-
		Pearson's R	0,672	0,869*	0,601	-
	HKU9	Spearman's Rho	0,788*	0,518	0,893**	0,929**
		Pearson's R	0,682	0,41	0,492	0,945**
	MERS-CoV (animal hosts)	Spearman's Rho	0,791*	-	-	-
		Pearson's R	0,774*	-	-	-
	MERS-CoV (human host)	Spearman's Rho	0,873*	-	-	-
		Pearson's R	0,883**	-	-	-
	MERS-CoV-related	Spearman's Rho	0,873*	-	-	-
		Pearson's R	0,780*	-	-	-
	Murine	Spearman's Rho	0,259	0,821*	0,857*	0,964**
		Pearson's R	0,446	0,566	0,536	0,971**
	PHEV	Spearman's Rho	0,316	-	0,893**	-
		Pearson's R	0,403	-	0,802*	-
	SARS-CoV	Spearman's Rho	-	-	0,927**	-
		Pearson's R	-	-	0,835*	-
	SARS-CoV-2	Spearman's Rho	0,630	-	-	-
		Pearson's R	0,511	-	-	-
	SARS-CoV-related	Spearman's Rho	0,883**	-	-	-
		Pearson's R	0,961**	-	-	-

Delta-coronavirus	HKU15	Spearman's Rho	1**	0,673	0,964**	-
		Pearson's R	0,995**	0,644	0,97**	-
Gamma-coronavirus	IBV	Spearman's Rho	0,857*	-	-	-
		Pearson's R	0,880**	-	-	-

Supplementary Table 2. Number of recombination breakpoints detected by RDP5 methods divided by species and genomic region. The percentages shown are calculated considering the total RB for each species. "N" represents the number of sequences given as input for RDP5. "Aggregate" is the sum of the data from all species. Values inside square brackets are the mean percentage of the genome alignments of the respective genomic region.

Species	Gene	3seq	%3seq	Geneconv	%Geneconv	RDP	%RDP	Bootscan	%Bootscan
hCoV-229E	*ORF1a*	3	25,0	9	17,3				
(N= 152)	*ORF1b*	5	41,7	19	36,5				
	S	0	0,0	15	28,8				
	E	0	0,0	0	0,0	-		-	
	M	0	0,0	0	0,0				
	N	1	8,3	0	0,0				
	Accessory	3	25,0	9	17,3				
hCoV-NL63	*ORF1a*	17	30,4	216	48,8				
(N=151)	*ORF1b*	17	30,4	220	49,7				
	S	14	25,0	2	0,5				
	E	0	0,0	0	0,0	-		-	
	M	0	0,0	0	0,0				
	N	1	1,8	0	0,0				
	Accessory	7	12,5	5	1,1				
HKU10	*ORF1a*	4	14,3	35	14,2	715	20,3	15	25,9
(N=25)	*ORF1b*	10	35,7	22	8,9	610	17,3	3	5,2
	S	9	32,1	62	25,2	427	12,1	8	13,8
	E	0	0,0	5	2,0	11	0,3	0	0,0
	M	1	3,6	31	12,6	117	3,3	2	3,4
	N	1	3,6	23	9,3	153	4,3	1	1,7
	Accessory	3	10,7	68	27,6	1487	42,2	29	50,0
HKU2	*ORF1a*	1	25,0	5	41,7	4	28,6	0	0,0
(N=10)	*ORF1b*	0	0,0	2	16,7	0	0,0	1	16,7
	S	2	50,0	1	8,3	3	21,4	2	33,3
	E	0	0,0	0	0,0	0	0,0	0	0,0
	M	0	0,0	1	8,3	0	0,0	0	0,0
	N	0	0,0	0	0,0	2	14,3	0	0,0
	Accessory	1	25,0	3	25,0	5	35,7	3	50,0
PEDV	*ORF1a*	43	22,2						
(N=851)	*ORF1b*	64	33,0						
	S	68	35,1						
	E	1	0,5	-		-		-	
	M	2	1,0						
	N	3	1,5						
	Accessory	13	6,7						
TGEV	*ORF1a*	4	25,0	1	25,0	1739	19,5	33	14,9
(N=62)	*ORF1b*	2	12,5	2	50,0	1127	12,7	24	10,8
	S	4	25,0	0	0,0	937	10,5	60	27,0
	E	0	0,0	0	0,0	54	0,6	4	1,8
	M	0	0,0	0	0,0	86	1,0	6	2,7

	N	1	6,3	1	25,0	903	10,1	7	3,2
	Accessory	5	31,3	0	0,0	4062	45,6	88	39,6
GCCDC1	*ORF1a*	1	10,0	4	14,3	16	11,1	4	13,3
(N=9)	*ORF1b*	2	20,0	4	14,3	6	4,2	2	6,7
	S	4	40,0	9	32,1	19	13,2	0	0,0
	E	0	0,0	3	10,7	10	6,9	2	6,7
	M	0	0,0	2	7,1	6	4,2	0	0,0
	N	0	0,0	0	0,0	17	11,8	0	0,0
	Accessory	3	30,0	6	21,4	70	48,6	22	73,3
hCoV-HKU1	*ORF1a*	2	50,0	44	44,0	2986	41,6	49	25,0
(N=63)	*ORF1b*	2	50,0	6	6,0	517	7,2	54	27,6
	S	0	0,0	2	2,0	903	12,6	14	7,1
	E	0	0,0	0	0,0	37	0,5	0	0,0
	M	0	0,0	0	0,0	106	1,5	2	1,0
	N	0	0,0	1	1,0	1397	19,4	2	1,0
	Accessory	0	0,0	47	47,0	1238	17,2	75	38,3
hCoV-OC43	*ORF1a*	3	11,5						
(N=342)	*ORF1b*	3	11,5						
	S	9	34,6						
	E	0	0,0	-		-		-	
	M	1	3,8						
	N	0	0,0						
	Accessory	10	38,5						
HKU4	*ORF1a*	0	0,0	7	23,3	7	16,7		
(N=10)	*ORF1b*	3	50,0	8	26,7	9	21,4		
	S	1	16,7	6	20,0	6	14,3		
	E	0	0,0	0	0,0	0	0,0	-	
	M	1	16,7	0	0,0	0	0,0		
	N	0	0,0	0	0,0	7	16,7		
	Accessory	1	16,7	9	30,0	13	31,0		
HKU9	*ORF1a*	3	50,0	4	23,5	52	17,4	0	0,0
(N=10)	*ORF1b*	2	33,3	4	23,5	49	16,4	2	20,0
	S	1	16,7	4	23,5	35	11,7	0	0,0
	E	0	0,0	0	0,0	0	0,0	0	0,0
	M	0	0,0	1	5,9	12	4,0	0	0,0
	N	0	0,0	4	23,5	21	7,0	1	10,0
	Accessory	0	0,0	0	0,0	129	43,3	7	70,0
MERS-CoV	*ORF1a*	0	0,0						
(animal hosts)	*ORF1b*	8	50,0						
(N=334)	*S*	8	50,0						
	E	0	0,0	-		-		-	
	M	0	0,0						
	N	0	0,0						
	Accessory	0	0,0						
	ORF1a	4	28,6	-		-		-	
	ORF1b	3	21,4						

MERS-CoV (human host) (N=286)	*S*	4	28,6						
	E	0	0,0						
	M	0	0,0						
	N	1	7,1						
	Accessory	2	14,3						
MERS-CoV-related (N=631)	*ORF1a*	3	13,6						
	ORF1b	4	18,2						
	S	9	40,9						
	E	0	0,0	-		-		-	
	M	1	4,5						
	N	1	4,5						
	Accessory	4	18,2						
Murine-CoV (N=38)	*ORF1a*	4	20,0	47	24,2	1584	18,2	15	8,9
	ORF1b	0	0,0	13	6,7	1650	19,0	19	11,3
	S	7	35,0	27	13,9	755	8,7	39	23,2
	E	0	0,0	1	0,5	69	0,8	1	0,6
	M	1	5,0	8	4,1	239	2,8	2	1,2
	N	0	0,0	12	6,2	702	8,1	3	1,8
	Accessory	8	40,0	86	44,3	3691	42,5	89	53,0
PHEV (N=18)	*ORF1a*	0	0,0			351	34,9		
	ORF1b	0	0,0			177	17,6		
	S	1	50,0			53	5,3		
	E	0	0,0	-		0	0,0	-	
	M	0	0,0			7	0,7		
	N	0	0,0			22	2,2		
	Accessory	1	50,0			396	39,4		
SARS-CoV (N=13)	*ORF1a*	0		0		19	43,2	0	
	ORF1b	0		0		22	50,0	0	
	S	0		0		1	2,3	0	
	E	0	-	0	-	0	0,0	0	-
	M	0		0		0	0,0	0	
	N	0		0		0	0,0	0	
	Accessory	0		0		2	4,5	0	
SARS-CoV-2 (N=2794)	*ORF1a*	6	33,3						
	ORF1b	2	11,1						
	S	4	22,2						
	E	0	0,0	-		-		-	
	M	2	11,1						
	N	2	11,1						
	Accessory	2	11,1						
SARS-CoV-related (N=276)	*ORF1a*	19	24,4						
	ORF1b	22	28,2						
	S	16	20,5						
	E	0	0,0	-		-		-	
	M	2	2,6						
	N	2	2,6						

	Accessory	17	21,8						
HKU15 (N=200)	*ORF1a*	16	20,0	6	18,8	32645	43,5		
	ORF1b	24	30,0	9	28,1	19919	26,6		
	S	20	25,0	7	21,9	7955	10,6		
	E	1	1,3	0	0,0	307	0,4	-	
	M	3	3,8	0	0,0	1215	1,6		
	N	4	5,0	6	18,8	5924	7,9		
	Accessory	12	15,0	4	12,5	7047	9,4		
IBV (N=667)	*ORF1a*	40	14,6						
	ORF1b	98	35,8						
	S	39	14,2						
	E	5	1,8						
	M	8	2,9						
	N	19	6,9						
	Accessory	65	23,7						
Aggregate (N=6942)	*ORF1a [43.7%]*	173	19,5	378	32,6	40118	38,3	116	16,8
	ORF1b [27.8%]	271	30,6	309	26,7	24086	23,0	105	15,2
	S [13.4%]	220	24,8	135	11,7	11094	10,6	123	17,8
	E [0.8%]	7	0,8	9	0,8	488	0,5	7	1,0
	M [2.3%]	22	2,5	43	3,7	1788	1,7	12	1,7
	N [4.6%]	36	4,1	47	4,1	9148	8,7	14	2,0
	Accessory [7.3%]	157	17,7	237	20,5	18140	17,3	313	45,4
Total		886	-	1158	-	104862	-	690	-
